# Structure of the DBL3X-DBL4ε region of the VAR2CSA placental malaria vaccine candidate: insight into DBL domain interactions

**DOI:** 10.1038/srep14868

**Published:** 2015-10-09

**Authors:** Stéphane Gangnard, Anita Lewit-Bentley, Sébastien Dechavanne, Anand Srivastava, Faroudja Amirat, Graham A. Bentley, Benoît Gamain

**Affiliations:** 1 Inserm UMR_1134, Paris, France; 2 Université Paris Diderot, Sorbonne Paris Cité, UMR_S1134 Paris, France; 3 Institut National de la Transfusion Sanguine, Paris, France; 4 Laboratory of excellence GR-Ex, Paris, France; 5 Unité d’Immunologie Structurale, Département de Biologie Structurale et Chimie, Institut Pasteur, 25 rue du Docteur Roux, 75724 Paris, France; 6 Centre National de la Recherche Scientifique URA2185, 25 rue du Docteur Roux, 75724 Paris, France

## Abstract

The human malaria parasite, *Plasmodium falciparum,* is able to evade spleen-mediated clearing from blood stream by sequestering in peripheral organs. This is due to the adhesive properties conferred by the *P. falciparum* Erythrocyte Membrane Protein 1 (PfEMP1) family exported by the parasite to the surface of infected erythrocytes. Expression of the VAR2CSA variant of PfEMP1 leads to pregnancy-associated malaria, which occurs when infected erythrocytes massively sequester in the placenta by binding to low-sulfated Chondroitin Sulfate A (CSA) present in the intervillous spaces. VAR2CSA is a 350 kDa protein that carries six Duffy-Binding Like (DBL) domains, one Cysteine-rich Inter-Domain Regions (CIDR) and several inter-domain regions. In the present paper, we report for the first time the crystal structure at 2.9 Å of a VAR2CSA double domain, DBL3X-DBL4ε, from the FCR3 strain. DBL3X and DBL4ε share a large contact interface formed by residues that are invariant or highly conserved in VAR2CSA variants, which suggests that these two central DBL domains (DBL3X-DBL4ε) contribute significantly to the structuring of the functional VAR2CSA extracellular region. We have also examined the antigenicity of peptides corresponding to exposed loop regions of the DBL4ε structure.

Most clinical manifestations of *Plasmodium falciparum* malaria arise from sequestration of parasitized erythrocytes (PEs) in diverse tissues, such as the microvasculature of different organs or the intervillous spaces of the placenta, as well as by adhesion to host cells, such as non-infected erythrocytes and platelets^1^. These cytoadhesion phenomena are mainly mediated by the *P. falciparum* erythrocyte membrane protein (PfEMP1) adhesin family, which is encoded by a family of roughly 60 *var* genes^2^. PfEMP1 is expressed on the surface of infected erythrocytes during the trophozoite stage, where the large antigenically variable extracellular region comprising several domains belonging to either the Duffy-binding like (DBL) or Cysteine-rich interdomain region (CIDR) protein folds mediates adhesion of PEs to host cell receptors such as CD36, ICAM1, EPCR and CSA^3^.

Pregnancy-associated malaria (PAM) results from the accumulation of PEs in the placenta *via* attachment to the glycosaminoglycan chondroitin sulphate A (CSA) present in the intervillous spaces^4^. VAR2CSA is the only member of the PfEMP1 family that has been associated with PAM^5^,^6^. Indeed, *var2csa* is the only *var* gene transcribed in placental isolates or CSA-binding laboratory strains and disruption of *var2csa* leads to the irreversible loss of CSA-binding phenotype^7^,^8^. Although VAR2CSA is polymorphic, women become immune to placental infections after one or more pregnancies by the acquisition of a protective humoral response in which antibodies that block CSA binding play a dominant role^9^,^10^,^11^,^12^. These antibodies recognize individual recombinant domains of VAR2CSA in a gender- and parity-dependent manner^13^ and, conversely, antibodies induced by recombinant VAR2CSA domains are surface-reactive with placental PEs^14^. Much interest has thus been devoted to developing VAR2CSA as a vaccine against PAM.

The extracellular region of VAR2CSA comprises six DBL domains (type ε or unknown (X)) and a CIDR (CIDRpam) module arranged in the following configuration^5^,^15^: DBL1X-DBL2X-CIDRpam-DBL3X-DBL4ε-DBL5ε-DBL6ε.

Although single recombinant domains have been shown to bind CSA^16^,^17^,^18^, recent data show that only the complete extra-cellular region of VAR2CSA fully reproduces the affinity and specificity of PEs expressing this variant^19^,^20^. Furthermore, analysis of the full-length VAR2CSA protein by small angle X-ray scattering (SAXS) demonstrated that it has a compact structure, probably due to well-defined interdomain interactions. This structural organization may thus be necessary to form the high-affinity, CSA-specific binding site, to which several domains contribute directly. Although the DBL2X domain in combination with the flanking interdomain regions shows high affinity binding similar to that of the full-length VAR2CSA^21^, only the DBL1X-DBL3X region exhibits the fine specificity for CSA^19^,^22^. This suggests that while DBL2X and flanking segments define an important region of the CSA-binding site, other domains also contribute by conferring specificity through additional contacts. Interestingly, the structure of PfEMP1 adhesin IT4VAR13, which binds to ICAM-1 via the DBL2β domain only, contrasts with the compact form of VAR2CSA^23^; here, SAXS analysis of IT4VAR13 shows an elongated structure where interdomain contacts appear to be confined largely to adjacent domains. Since an important component of immune protection against placental PEs arises from blocking adhesion to CSA, defining the binding site in atomic detail should contribute to optimization of vaccines based on VAR2CSA. This can be achieved by determining the crystal structures of individual or multiple domains. Until now, only DBL3X and DBL6ε structures have been solved^24^,^25^,^26^.

We have embarked on a structural study of VAR2CSA multidomain constructs in order to analyze the structural organization of the domains in detail. Here we report the crystal structure of the DBL3X-DBL4ε double domain from the FCR3 strain. The structure of the FCR3-DBL3X domain has already been described^24^,^25^; however we report here for the first time the crystal structure of DBL4ε, the least polymorphic domain of VAR2CSA as well as a detailed description of the contact interface between those two domains^27^. Of particular note, some novel features in the DBL motif have been identified. Contacts between the DBL3X and DBL4ε domains in the crystal structure are made essentially by invariant or highly conserved residues, suggesting that these also occur in the full-length protein and contribute to its compact organization. Although DBL4ε does not contribute to the binding site^28^, it induces antibodies that inhibit the binding of placental PEs to CSA^29^. These antibodies are PEs surface-reactive and epitope mapping has revealed dominant antigenic regions^30^. With the support of the DBL4ε structure, we have examined the antigenicity of a number of loop regions of the domain and analyzed the results in the light of other studies.

## Results

### Description of the FCR3-DBL3X-DBL4ε structure

**Figure 1:**
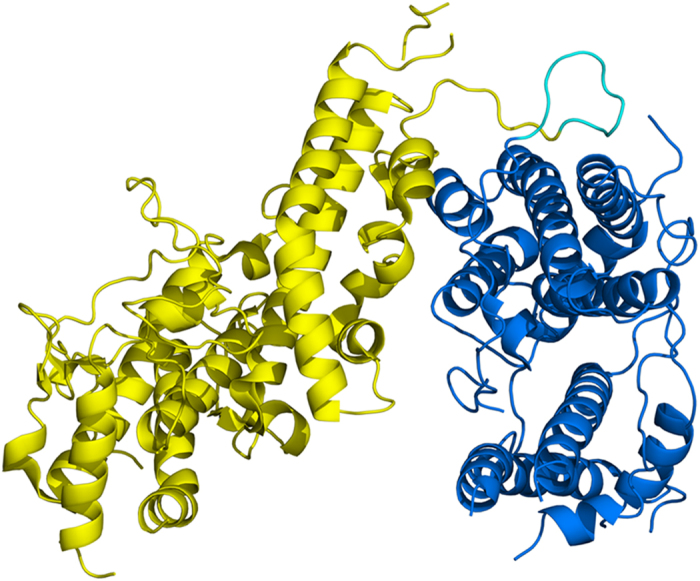
Structure of the FCR3-DBL3X-DBL4ε double domain. The structure is shown in ribbon representation with DBL3X in yellow and DBL4ε in blue. The interdomain linker between residues K1583 and K1590 inclusive is in cyan.

**Figure 2:**
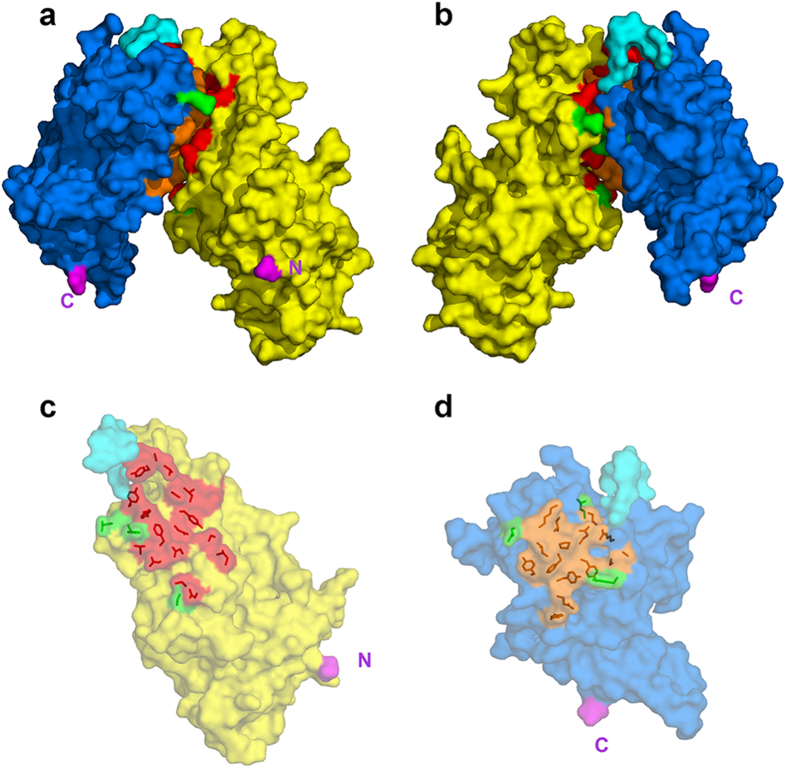
Interface between the DBL3X and DBL4ε domains. The domains are shown in surface representation with DBL3X in yellow, DBL4ε in blue and the linker region in cyan. The invariant contacting residues (see Table S1) are in red for DBL3X and orange for DBL4ε; polymorphic contacting residues are green for both domains. The N- and C-termini of the double domain are in magenta and are labelled N and C. (**a**,**b**) The DBL3X-DBL4ε double domain viewed edge on from the interdomain interface. The two views are rotated by 180° with respect to each other about the vertical (**c**) Semi-transparent surface representation of the DBL3X domain viewed from above the interface with the side chains of the contacting residues shown in red. (**d**) Semi-transparent surface representation of the DBL4ε domain viewed from above the interface with the side chains of the contacting residues shown in orange.

Expression of the recombinant FCR3-DBL3X-DBL4ε double domain has been previously described^22^. The recombinant protein includes residues S1215 to M1950 of VAR2CSA from the FCR3 laboratory strain (GeneBank entry AY372123), together with the four-residue sequence MASM arising from the NheI cloning site at the N-terminus and a C-terminal hexa-histidine tag to facilitate purification. The structure was solved and refined at 2.9 Å resolution (Table 1). The DBL3X moiety was oriented and positioned by molecular replacement using the FCR3-DBL3X structure (PDB entry 3BQK) as a search model, while the DBL4ε domain was built progressively into the electron density maps during refinement. The polypeptide chain of the refined structure could be traced from S1218 to V1922 with gaps Q1386-T1398 (DBL3X), N1479-K1485 (DBL3X), G1492-Q1493 (DBL3X), E1614-R1617 (DBL4ε), A1746-G1750 (DBL4ε), D1822-N1835 (DBL4ε), N1871-S1881 (DBL4ε) and N1923-M1950 (DBL4ε). Both domains display the helical secondary structure and the pattern of canonical cysteines (Fig. S1a and b) characterizing the DBL motif. The double domain forms a compact structure with a short linker (interdomain) region connecting the two DBL modules (Fig. 1). A total of 19 residues separate the last canonical cysteine of DBL3X, C1576 or canonical Cys(12) (the n-th canonical cysteine of a DBL domain is denoted by Cys(n)) and the first of DBL4ε (C1596, Cys(1)). The ten residues, K1583 to K1592, in the central region of the peptide connecting the two domains are significantly more variable in sequence in comparison to the flanking regions, which show the more conserved sequence pattern characteristic of constant blocks of VAR2CSA domains^15^. We have therefore defined this segment as the interdomain linker. The DBL3X-DBL4ε interface is extensive and includes 18 polar interactions between residues that are largely conserved in sequence (Fig. 2, Table S1). The polymorphic residues making contacts at the interface (3 out of 22 for DBL3X and 4 out of 16 for DBL4ε; see Table S1) are located at the periphery where stereochemical accommodation of the different polymorphs is easier than in the more buried central regions (Fig. 2c,d). A total surface accessible area of 1340 Å^2^ is buried at the interface between DBL3X and DBL4ε.

The structure of the FCR3-DBL3X single domain has been previously published^24^,^25^ (PDB entries 3CML and 3BQK, respectively). The DBL3X domain in the double domain reported here superimposes on the 3CML and 3BQK structures with an r.m.s.d. in the Cα positions of 2.67 Å and 2.29 Å, respectively. The larger difference of DBL3X in the double domain structure with respect to the two single domain structures (3CML and 3BQK superimpose upon each other with a 1.66 Å r.m.s.d. in Cα positions) arises from a large difference in conformation of the loop between C1251 (Cys(2)) and C1264 (Cys(3)); the r.m.s.d. reduces to 1.45 Å and 1.63 Å with the FCR3-DBL3X structures 3CML and 3BQK, respectively, when this region is removed from the superposition. This structural difference is caused by contacts with symmetry-related molecules in this region for the double domain structure.

The structure of DBL4ε, the least polymorphic VAR2CSA domain, has not been previously described; however, the pattern of DBL secondary structure is generally preserved (Fig. S1a). As in other DBL structures, Subdomain 1 possesses little regular secondary structure; the short conserved α-helix αH1 that is associated with the PPRX motif present in all DBL domains and an additional α-helix αH1′ is located in its C-terminal region. Subdomain 2 contains the conserved four-helix bundle (αH2, αH3, αH4 and αH5) but, unlike other DBL domains, αH5 possesses an 11-residue insertion (N1744-K1754). Subdomain 3 has the two long helices αH6 and αH7, characteristic of this region, and terminates with three short helices αH7, 3_10_H9 and αH9.

### Antigenicity of loop regions from the DBL4ε domain

**Figure 3:**
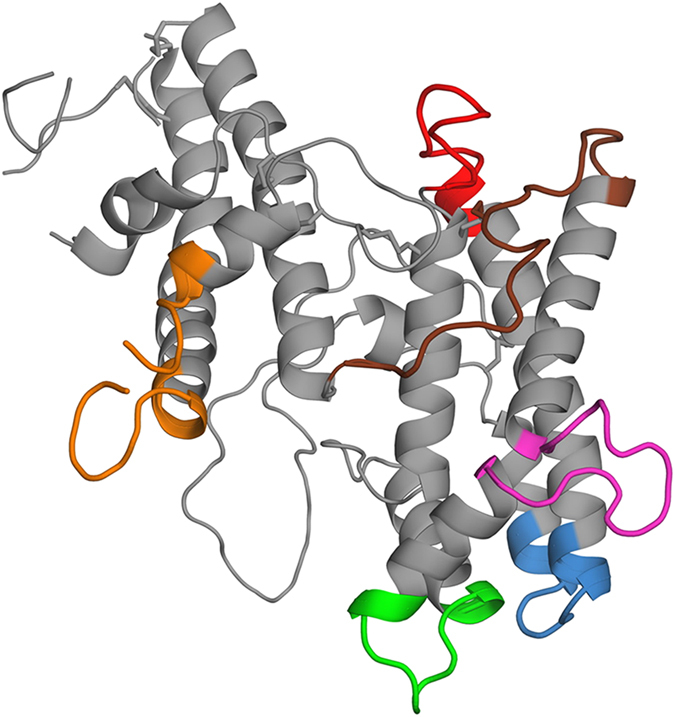
Peptide segments used in antigenicity studies of antibodies raised to the DBL3X-DBL4ε recombinant double domain. The peptides, chosen from exposed loop regions as described in the main text, are shown in their structural context. Colour code: Pep-1, Ile1640-Lys1651 (IIKNEEGMEKAK), blue; Pep-2, Gln1672-Lys1685 (QYNPTGKGIDDANK), red; Pep-3, Glu1722-Asp1733 (EIFGSSDTNDID), green; Pep-4, Glu1743-ILe1756 (ENETITNGPDRKTI), mauve; Pep-5, Glu1774-Lys1792 (EEKNENFPL**S**MGVEHIGIAK)*, brown; Pep-6, Asn1864-Asp1890 (NKIYRKSNKESEGGKDYSMIMAPTVID), orange. *Underlined residue in Pep-5 was changed from Cys in the cognate sequence to Ser in order to avoid cross-linking of peptides.

As our study provided the first experimental structure of the DBL4ε VAR2CSA module, we examined the structural context of antigenic regions in this domain. Accordingly, we assessed the antibody response of a rabbit, immunized with the DBL3X-DBL4ε double domain, to a selected set of DBL4ε peptides. We selected peptide sequences corresponding to exposed loop regions that connect the conserved helices of the DBL fold. Some segments in Subdomain 3, however, were excluded since certain regions could not be traced in the electron density maps or because the presence of several cysteine bridges might compromise structural mimicry of these regions by peptides. Using these criteria, we selected six polypeptide segments denoted Pep-1 to Pep-6, which are detailed in Fig. 3. Peptides were synthesized with a biotin moiety attached to the N-terminus by a linker comprising two glycine residues and ε-aminohexanoic acid. We reasoned that this should allow optimal exposure of the DBL4ε peptides with minimal steric hindrance and thus favour their capture on streptavidin-coated magnetic beads or by the anti-biotin mouse monoclonal antibody^31^.

**Figure 4:**
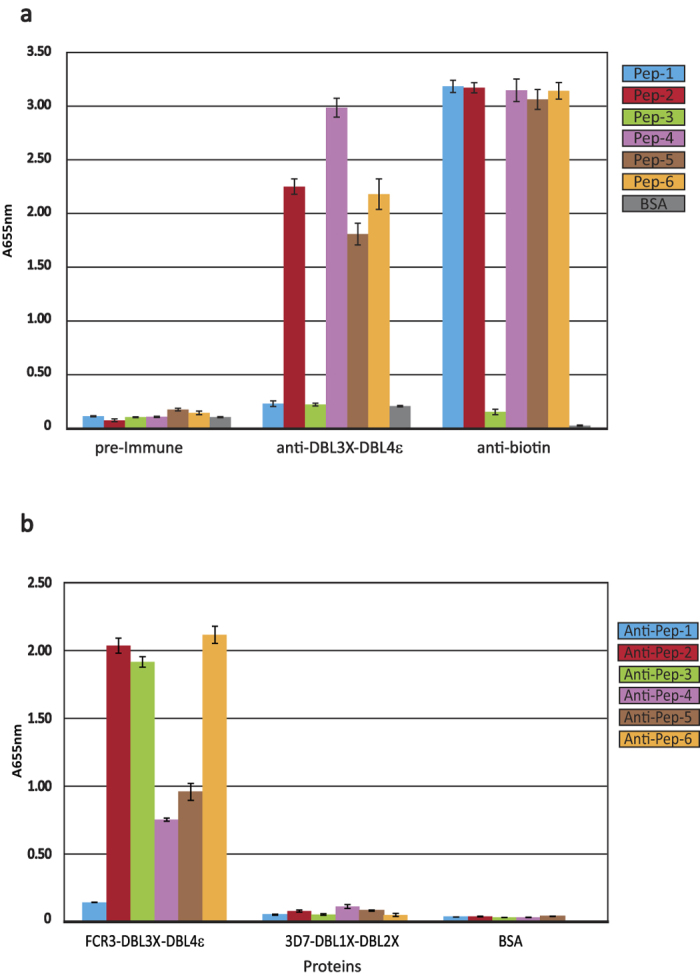
Reactivity of DBL4ε-derived peptides and FCR3-DBL3X-DBL4ε with purified anti- DBL3X-DBL4ε rabbit IgG. **(a)** Recognition of peptides (Pep-1 to Pep-6) by anti-DBL3X-DBL4ε purified IgG antibodies. Biotinylated peptides were directly coated in carbonate buffer. ELISA was performed using the pre-Immune IgG (10 μg/mL), the anti-DBL3X-DBL4ε IgG (10 μg/mL) or the anti-biotin Mouse monoclonal IgG (1 μg/mL). (**b**) Recognition of recombinant FCR3-DBL3X-DBL4ε by immune IgG purified on peptides, named Anti-pep1 to 6. The 3D7-DBL1X-DBL2X double domain and Bovine Serum Albumin (BSA) were used as negative controls. The optical density was measured at 655 nm. All proteins were coated at 1 μg/mL and purified rabbit IgG was used at a dilution of 1:100 in the blocking buffer.

Antigenicity of the peptides was examined with protein G-purified IgG from a rabbit immunized with the recombinant FCR3-DBL3X-DBL4ε protein. Two different experimental protocols were followed to assess consistency of the results. The first approach used solid phase ELISA, where the peptides were coated onto 96-well plates and reacted with the purified IgG (Fig. 4a). Pre-immune antibodies were used as a negative control and the presence of coated peptide was verified using an anti-biotin mAb. Pep-2, Pep-4, Pep-5 and Pep-6 were detected with the purified immune IgG but no reaction was observed with the pre-immune antibodies, showing that these peptides were specifically recognized by the anti-FCR3-DBL3X-DBL4ε IgG. Pep-1 and Pep-3 showed no reaction with either the immune or pre-immune antibodies. All peptides, except Pep-3, were shown to be coated as they were detected with the anti-biotin mAb. Thus Pep-3 was either not coated on the plates or, was adsorbed in such a way that the biotin moiety was not accessible. These results indicate that peptides Pep-2, Pep-4, Pep-5 and Pep-6 are antigenic.

In the second experimental protocol, peptide-specific antibodies were purified by binding the immune IgG to the peptides immobilized on streptavidin-coated magnetic beads. After elution from the beads, antibodies from each of the peptides were tested in turn by ELISA for reactivity with the FCR3-DBL3X-DBL4ε immunogen, as well as with 3D7-DBL1X-DBL2X and Bovine Serum Albumin negative controls (Fig. 4b). Consistent with the peptide ELISA results, Pep-2, Pep-4, Pep5 and Pep-6 retained IgG that recognized the recombinant double domain but not the control proteins. Moreover, consistent with the absence of reactivity observed by ELISA on the peptides, no specific IgG was eluted from the immobilized Pep-1. By contrast, immobilized Pep-3 yielded IgG that reacted strongly with FCR3-DBL3X-DBL4ε, showing that the peptide was indeed antigenic. The negative result for Pep-3 in the peptide ELISA (Fig. 4a) was thus probably due to its poor presentation when coated onto the plastic surface.

We therefore conclude that peptides Pep-2, Pep-3, Pep-4 Pep-5 and Pep-6 are antigenic. More importantly, the fact that immune IgG purified on each of these peptides reacted with the recombinant FCR3-DBL3X-DBL4ε confirms that the corresponding regions on the DBL4ε structure are also antigenic.

## Discussion

Our study has aimed to extend structural knowledge of the individual VAR2CSA domains and to contribute to understanding of the interdomain organization of its extracellular region. The structure of the double domain FCR3-DBL3X-DBL4ε provides the first structural description of the DBL4ε module, which is the least polymorphic domain of VAR2CSA, and gives a detailed atomic view of the interdomain interactions between the two central domains of this variant. The double domain forms a compact structure with a short linker of ten residues, K1583 to K1592 (interdomain) region connecting the two DBL modules (Fig. 1). The interface between the two domains is highly conserved and is significantly polar as it includes 18 hydrogen bonds. Indeed, contacts between the DBL3X and DBL4ε domains in the crystal structure are made essentially by invariant or highly conserved residues (Fig. 2, Table S1), suggesting that these contacts also occur in the full-length VAR2CSA and contribute to its compact organization^19^,^20^.

The three C-terminal domains of VAR2CSA belong to the ε class of the PfEMP1-DBL fold. The ε class is typified, in particular, by the absence of canonical cysteines Cys(2) and Cys(3), which form a disulfide bridge in other DBL classes, and the presence of Cys(10b) and Cys(10c), which form a bridge. Moreover, Cys(10c) always precedes Cys(10a) by two residues^3^,^15^. Nonetheless, DBL4ε, DBL5ε and DBL6ε, each separately, present unique features that are preserved within these respective domains of VAR2CSA. Crystal structures have been determined for DBL4ε (this work) and DBL6ε^26^,^32^.

### (a) DBL4ε

Our study provides the first structure determination of a VAR2CSA DBL4ε domain. The canonical Cys(-1)-Cys(5a) disulfide bridge, linking subdomains 1 and 2 of PfEMP1-DBL domains, is absent in FCR3-DBL4ε structure; this absence is characteristic of the DBL4ε-VAR2CSA domain in other VAR2CSA variants^15^. Residue S1766, which aligns with Cys(5a) of other DBL domains in both sequence and structural alignments, is invariant in DBL4ε-VAR2CSA^15^. A novel disulfide bridge, however, is observed between C1689 and C1906, connecting Subdomains 1 and 2 of DBL4ε-FCR3. These residues are present in all DBL4ε-VAR2CSA sequences^15^ and we denote these as canonical Cys(5′) and Cys(10′), respectively. The loop between Cys(10b) and Cys(10c) (C1896 and C1907, respectively, in DBL4ε-FCR3) is surface-exposed and is highly conserved, carrying the sequence (N/H)GEINGNYI^15^. DBL4ε-FCR3 lacks the pattern of the two C-terminal canonical cysteines, Cys(11) and Cys(12), separated by one residue, which is typical of all other DBL types (except DBL1α)^15^,^33^. Indeed, we were uncertain of the C-terminal limit when designing the recombinant construct because of this absence in DBL4ε. Interestingly, canonical Cys(8) and Cys(10), which form disulfide bridges with Cys(12) and Cys(11), respectively, in the DBL motif, are present in DBL4ε-FCR3 (C1830 and C1842, respectively). Three cysteine residues, C1933, C1961 and C1991, occur between the last observed cysteine in the DBL4ε structure (C1909, canonical Cys(10a)) and the first cysteine of DBL5ε (C2001, canonical Cys(-1)); these are C1933, C1961 and C1991. These cysteines are conserved in DBL4ε-VAR2CSA sequences^15^. Our construct terminated at D1941 and thus included C1933 only; we were unable to trace this residue in the electron density maps.

In all known structures of DBL domains, whether from PfEMP1 or erythrocyte-binding proteins (EBA-175, DBP), helix αH5 contains a conserved motif WWX_7_W, where this helix is kinked at the 4th residue. In DBL4ε-FCR3, however, the interval between the conserved tryptophan residues comprises 18 amino acids (WWX_18_W), of which 11 form a loop with no regular secondary structure. This loop is surface exposed and does not interact with the DBL3X domain. In different DBL4ε-VAR2CSA domains, the distance between the conserved tryptophan residues varies between 18 to 25 amino acids and the region is highly variable in sequence, except at the N- and C-terminal ends; this would correspond to an insertion of a loop comprising 11 to 18 residues into the αH5 helix in the different VAR2CSA strains. This region has been previously noted as a variable block region unique to the DBL4ε domain^34^.

### (b) DBL5ε

Since the structure of DBL5ε has not yet been determined, the discussion is restricted to sequence analysis only. Canonical Cys(5) and Cys(6) are disulfide bridged in the DBL motif; however, while Cys(6) is present in all known DBL5ε-VAR2CSA sequences (C2171 in FCR3), Cys(5) is absent. The loop between Cys(10b) and Cys(10c) is only two residues long and is quite well conserved in sequence^15^. Cys(10a) is the last canonical cysteine of DBL5ε, since it lacks Cys(11) and Cys(12) (which form cysteine bridges to Cys(10) and Cys(8), respectively). Cys(8) is also absent in all known VAR2CSA DBL5ε sequences.

### (c) DBL6ε

Crystal structures of DBL6ε from the FCR3 and 3D7 laboratory strains have been solved^26^,^32^ and the two structures are compared in detail elsewhere^32^. In both, Cys(1) and Cys(4) are absent in all known VAR2CSA sequences and this canonical disulfide bridge is thus not formed. A cysteine, denoted Cys(4′) (C2383 in FCR3)^32^, is unique to DBL6ε and is present in all sequences. This does not form a disulfide bridge within the DBL6ε domain but we do not exclude the possibility of an interdomain bridge involving this residue. Cys(5) is absent in all DBL6ε-VAR2CSA sequences but Cys(6) is present in some; for example it is present in DBL6ε-FCR3 (C2493) but not in DBL6ε-3D7. Cys(10b) and Cys(10c) are separated by a segment that is two or four residues long and is variable in sequence; Cys(10b) and Cys(10c) is absent in some DBL6ε sequences.

Several studies analysing the immune response to VAR2CSA have demonstrated that DBL4ε-specific antibodies are able to label and inhibit the adhesion of *P. falciparum* infected erythrocytes in a strain-independent manner, showing that this domain is surface-exposed in the full-length VAR2CSA^29^,^35^,^36^,^37^. Nanobodies raised against the full-length VAR2CSA, but with specificity to DBL4ε domains, were also found to be surface-reactive although not inhibitory^38^,^39^. These data and the fact that DBL4ε does not participate in the minimal binding region of VAR2CSA^21^,^22^, suggest that inhibition could involve steric hindrance and/or abolition of the native higher-order domain organisation. Our structural results led us to explore the antigenicity of exposed regions of DBL4ε using protein G-purified IgG obtained by immunization of a rabbit with the recombinant double domain. Peptides with sequences derived from six different exposed loops of the DBL4ε domain were synthetized and tested for antigenicity using anti FCR3-DBL3X-DBL4ε polyclonal IgG. Our results demonstrate that the regions on the domain represented by Pep-2, Pep-3, Pep-4, Pep-5 and Pep-6 are antigenic as the peptides are specifically recognized by the immune IgG and antibodies purified on these peptides react with the recombinant double domain (Fig. 4b). Pep-1, by contrast, is not antigenic (Fig. 4a,b). While it may seem very likely that the region of the DBL4ε domain corresponding to Pep-1 is not antigenic either, we cannot exclude the possibility that the peptide showed no response to the immune IgG because it is a poor conformational mimic of this structured region of the native protein. We note, however, that a previous epitope mapping study^30^ using several different peptides encompassing the Pep-1 sequence were also not reactive to antibodies, reinforcing the conclusion that this region of the native protein is indeed unlikely to be antigenic. Interestingly, this region is in close proximity to DBL3X in the double domain, which could affect its accessibility to antibodies. Pep-5 and Pep-6 correspond to more conserved regions of DBL4ε than the other peptides^15^, suggesting that in the context of the full-length VAR2CSA these regions might be less exposed to the immune response.

Our results correlate in part with previous pepscan mapping and B-cell epitope prediction of the FCR3-DBL4ε sequence using rat and human immune antibodies^30^. The Pep-2 and Pep-6 sequences used in our study comprise complete B-cell epitopes identified by Ditlev *et al.*^30^ while the Pep-3 and Pep-4 sequences are respectively, the N-terminal and the C-terminal sequences of another predicted B-cell epitope. In the structure, Pep-3 and Pep-4 are separated by the N-terminal section of the kinked α-helix αH5 and Pep-4 corresponds to the inserted loop of αH5 that is unique to DBL4ε. While peptides encompassing Pep-2 and Pep-4 were highly recognized by anti-DBL4ε rat sera, low reactivity was observed for peptides encompassing Pep-5 and Pep-6^30^. Contrary to our study, Ditlev *et al.*^30^ found that a peptide (P34) encompassing Pep-3 was not antigenic. It is highly probable that this negative result in the Pepscan is due to its poor presentation when coated onto the plastic surface, as observed in our study (Fig. 4a). However, the absence of antigenicity of sequences including Pep-1 is concordant with our findings. Importantly, in this study the authors narrowed down a region of DBL4ε that is targeted by inhibitory anti-DBL4ε IgG and is also an important target during PAM^30^. This region, ranging from W1670 to S1694, belongs to a predicted B-cell epitope and encompasses the Pep-2 segment. By mapping this region on a predicted structural model, the authors estimated its location around a highly conserved loop flanked by two small helices in the S2-subdomain. However, when mapped on our DBL4ε structure, this region displays a loop formed by 6 residues only (N1674 to G1679), which is significantly smaller than the 11 residues proposed in the model (N1674 to G1684) (Figs S1a and S2). As canonical Cys(5) and Cys(5′) present in the second helix of this region are engaged in disulfide bonds with respectively Cys(6) and Cys(10′) (Fig. S1b), this could explain why peptides designed by Ditlev *et al.* to cover this loop region and used to immunize rats, failed to elicit an efficient immune response against the native VAR2CSA expressed on infected erythrocytes.

Our crystallographic study of the FCR3-DBL3X-DBL4ε double domain is a first step towards providing atomic detail of the domain organisation of the extracellular region of VARCSA. It has revealed an interface between the DBL3X and DBL4ε domains that is highly conserved within VAR2CSA variants (Fig. 2, Table S2) and is thus likely to be preserved in the full length VAR2CSA structure. The configuration of this domain pair quite likely contributes to the compact organization of the full-length VAR2CSA observed by SAXS^19^,^20^. Similar crystallographic studies with other domain combinations could therefore hold promise of defining the interdomain interfaces and thus building a three-dimensional structure of this complex, polymorphic, multidomain region of VAR2CSA. Such information will be invaluable for identifying and understanding the nature of the CSA-binding site and regions that are targeted by inhibitory antibodies. In turn, these data should greatly aid the design and development of efficient vaccines that block the interaction between VAR2CSA and the placenta.

## Methods

### Ethics statement

All animal vaccination experiments were executed in strict accordance with good animal practices, following the EU animal welfare legislation and after approval of the Biotem and INSERM ethical committees. Every effort was made to minimize suffering.

### Expression and purification of recombinant FCR3-DBL3X-DBL4ε

Expression of the recombinant FCR3-DBL3X-DBL4ε double domain has been previously described^22^. The synthetic gene, from which DBL3X-DBL4ε DNA was amplified, carried mutations at potential N-glycosylation sites to allow production of the recombinant protein in eukaryotic, as well as bacterial, expression systems. N-glycosylation mutations were as follows: N1222Q, N1290Q, S1430G, S1594L, T1746A, T1751P, T1846A, N1916Q. The protein was expressed in *E. coli* SHuffle^®^ strain of (Novagen). Cells were centrifuged and resuspended in lysis buffer (20mM Tris-HCl, 150 mM NaCl, pH8) and lysis was performed with an Emulsiflex homogeniser (Avestin). Lysate was cleared by centrifugation at 8000 g during 1 h. FCR3-DBL3X-DBL4ε was first captured on a heparin column and eluted with 20mM Tris-HCl, 1M NaCl, pH8. The eluate was loaded on a metal affinity column (TALON, Clontech) and FCR3-DBL3X-DBL4ε recovered with 20 mM Tris-HCl, 150 mM NaCl, 200 mM Imidazole, pH8. A final purification step to remove possible impurities and aggregates was carried out by gel filtration (S75 16/60, GE Healthcare). In order to improve crystal quality, the protein was subsequently subjected to an additional purification step on a CM-Sepharose column (GE Healthcare) equilibrated in 20 mM HEPES pH 7, 40 mM NaCl. The protein was eluted by increasing NaCl concentration by 5% (w/v) steps and concentrated to 9 mg/ml.

### Crystallization

Crystals were grown using the hanging-drop vapour-diffusion technique by mixing 1 μL protein solution with 0.75 or 1 μL reservoir solution and equilibrating against 1 mL reservoir solution. Initial crystallisation conditions were identified using robotic screening with several commercial crystallisation screens at the Crystallography Platform of the Pasteur Institute. These conditions were further refined by manual experiments using the PEG-Ion screen (Hampton). The best crystals were grown in 18% PEG 3000, 100 mM Bis-Tris buffer, pH 6.5, 300 mM NaCl and 10% v/v glycerol for the first data-quality crystal, none for the second one. Crystals were mounted on cryoloops, rapidly passed through 15% glycerol and flash-frozen in liquid nitrogen for data collection.

### Data collection, processing and structure determination

Native data were collected on the ID29 beamline at the ESRF, Grenoble, France and on Proxima1 at SOLEIL, St. Aubin, France. All data were treated with XDS^40^, followed by SCALA from the CCP4 programme suite^41^. Since diffraction was anisotropic, a variable high resolution cut-off ranging from 2.9 Å to 3.3 Å was applied as a function of crystal orientation in order to achieve a satisfactory scaling of the data frames. The highest resolution shell is thus only complete to 50%. Crystal parameters and data collection statistics are detailed in Table 1(a).

The structure determination was initiated with molecular replacement using AMoRe^42^ and the high-resolution structure of DBL3X (PDB ID 3BQK^24^, as a search model. Programs Molrep and FFEAR from the CCP4 programme suite were used to locate helices that would correspond to the DBL4ε domain. The structure of DBL6ε (PDB ID 2Y8D), overlaid upon these helices, was used to help with the tracing of the polypeptide chain. The structure was refined through several cycles using the program Buster (Global Phasing Ltd, Cambridge, England), combined with model rebuilding using Coot^43^. The final refinement statistics are presented in Table 1(b)^44^. Although the diffraction was weak, we found it important to include intensities to 2.9 Å resolution. As has been underlined elsewhere^45^, these highest resolution data were essential in helping to resolve certain less clear regions of the structure in the electron density maps. Portions of Electron Density Map are shown in Figure S3. The coordinates and diffraction data have been deposited in the Protein Data Bank (PDB entry 4P1T).

### Production of anti-FCR3-DBL3X-DBL4ε antibodies

The anti-FCR3-DBL3X-DBL4ε rabbit IgGs used in this study were produced by immunization of a New Zealand white rabbit. Immunization with the recombinant double domain was performed by Biotem, France, according to animal immunization guidelines. In brief, a NZ white rabbit received intradermally 40 μg of recombinant protein in Freund’s Complete Adjuvant for first immunization and 20 μg subcutaneously for three boosts. Antisera were collected 63 days after the first injection. IgG were purified from sera by affinity chromatography on protein G.

### Purification of antibodies on peptides

Peptides corresponding to exposed loop regions of the DBL4ε domain (Fig. 3) were provided by Genecust (Luxembourg). A biotin moiety was attached to the N-terminus of each peptide *via* a linker comprising ε-aminohexanoic acid (Ahx) and two glycine residues. This allowed fixing a constant quantity of each peptide to streptavidin-coated beads and minimized potential steric hindrance, favouring more efficient antibody recognition. A quantity of 2.6 × 10^−8^ mol of each biotinylated peptide was incubated with 200 μL streptavidin-coated magnetic beads (Dynabeads Myone Streptavidin T1; Invitrogen) and protein G-purified antibodies from a rabbit immunized with FCR3-DBL3X-DBL4ε were affinity purified on these peptides. 250 μg of immune or pre-immune protein G-purified antibodies diluted in PBS were incubated with 200μL beads (capacity 0.4 × 10^−9^ mol of peptides/mL), pre-equilibrated with PBS. After extensive washing of the beads with PBS, antibodies bound to the beads were eluted with 100 μL elution buffer (0.1 M glycine, pH 2.7). The eluate was neutralized with 20 μL of neutralizing buffer (1 M Tris, pH 9).

### Responses of purified antibodies to recombinant FCR3-DBL3X-DBL4ε and peptides

Peptide-purified IgG was tested either on the recombinant domain or on the biotinylated peptides. ELISA was performed on recombinant proteins as follows. Recombinant FCR3-DBL3X-DBL4ε or 3D7-DBL1X-DBL2X (used as a negative control) was coated overnight at 4 °C on 96-well plates at a concentration of 1 μg/mL. Wells were coated with blocking buffer (1% bovine serum albumin and 0.05% Tween 20 in PBS) during 1 h at 37 °C. After three further washing steps, wells were incubated with peptide-purified IgG diluted in blocking buffer (1:100) for 1 h at room temperature. IgG were revealed with horseradish-peroxidase-conjugated Ig anti-rabbit IgG (1:200 in blocking buffer), using TMB as a substrate. ELISA was performed on immobilized peptides as follows. Biotinylated peptides were immobilized on 96-well plates at 200 μg/mL in 50 mM carbonate buffer pH 9.6, overnight at 4 °C. After a blocking step, affinity-purified antibodies (dilution 1:100) or an anti-biotin Mouse monoclonal antibody (Jackson Immunoresearch, UK) were added and incubated for 1 h at 37 °C. After extensive washing, horseradish-peroxidase-conjugated Ig anti-rabbit IgG (1:2000) was incubated 1 h at room temperature. TMB was used as a substrate and absorbance measured at 655 nm.

## Additional Information

**How to cite this article**: Gangnard, S. *et al.* Structure of the DBL3X-DBL4ε region of the VAR2CSA placental malaria vaccine candidate: insight into DBL domain interactions. *Sci. Rep.* **5**, 14868; doi: 10.1038/srep14868 (2015).

## Supplementary Material

Supplementary Information

## Figures and Tables

**Table 1:**
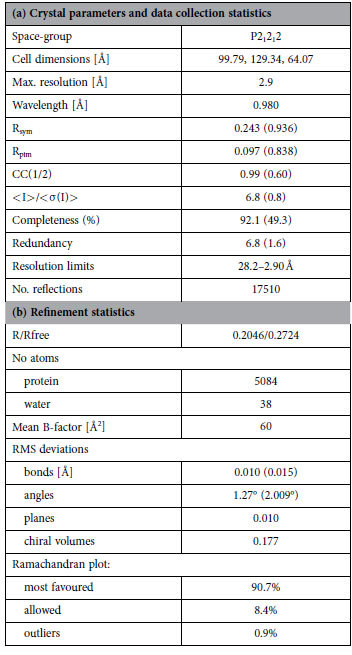
Crystallographic data.

R = Σ|F_o_ – F_c_|/ΣF_o_, 5% exclusion was used for R_free_. The percentage of peptide bonds in the most favoured and additional allowed region of the Ramachandran plot was determined by RAMPAGE^44^.
