# Supplementary material for: Structure of the DBL3X-DBL4ε region of the VAR2CSA placental malaria vaccine candidate: insight into DBL domain interactions
Source: Sci Rep. 2015 Oct 9;5:14868. doi: 10.1038/srep14868 (PMC4598876; doi:10.1038/srep14868)
Supplement: Supplementary Information [file srep14868-s1.doc]

**Structure of the DBL3X-DBL4ε region of the VAR2CSA placental malaria vaccine candidate: insight into DBL domain interactions**

**Stéphane Gangnard*†‡§∥¶, Anita Lewit-Bentley∥¶, Sébastien Dechavanne*†‡§, Anand Srivastava*†‡§, Faroudja Amirat∥¶, Graham A. Bentley∥¶^1^, Benoît Gamain*†‡§^1^**

*****Inserm UMR_1134, Paris, France; Université Paris Diderot

**†** Sorbonne Paris Cité, UMR_S1134 Paris, France

**‡** Institut National de la Transfusion Sanguine, Paris, France

**§** Laboratory of excellence GR-Ex, Paris, France,

**∥** Unité d'Immunologie Structurale, Département de Biologie Structurale et Chimie, Institut Pasteur, 25 rue du Docteur Roux, 75724 Paris, France,

**¶** Centre National de la Recherche Scientifique URA2185, 25 rue du Docteur Roux, 75724 Paris, France

**^1^** To whom correspondence should be addressed: (email benoit.gamain@inserm.fr, bentley@pasteur.fr).

**Supplementary**

**
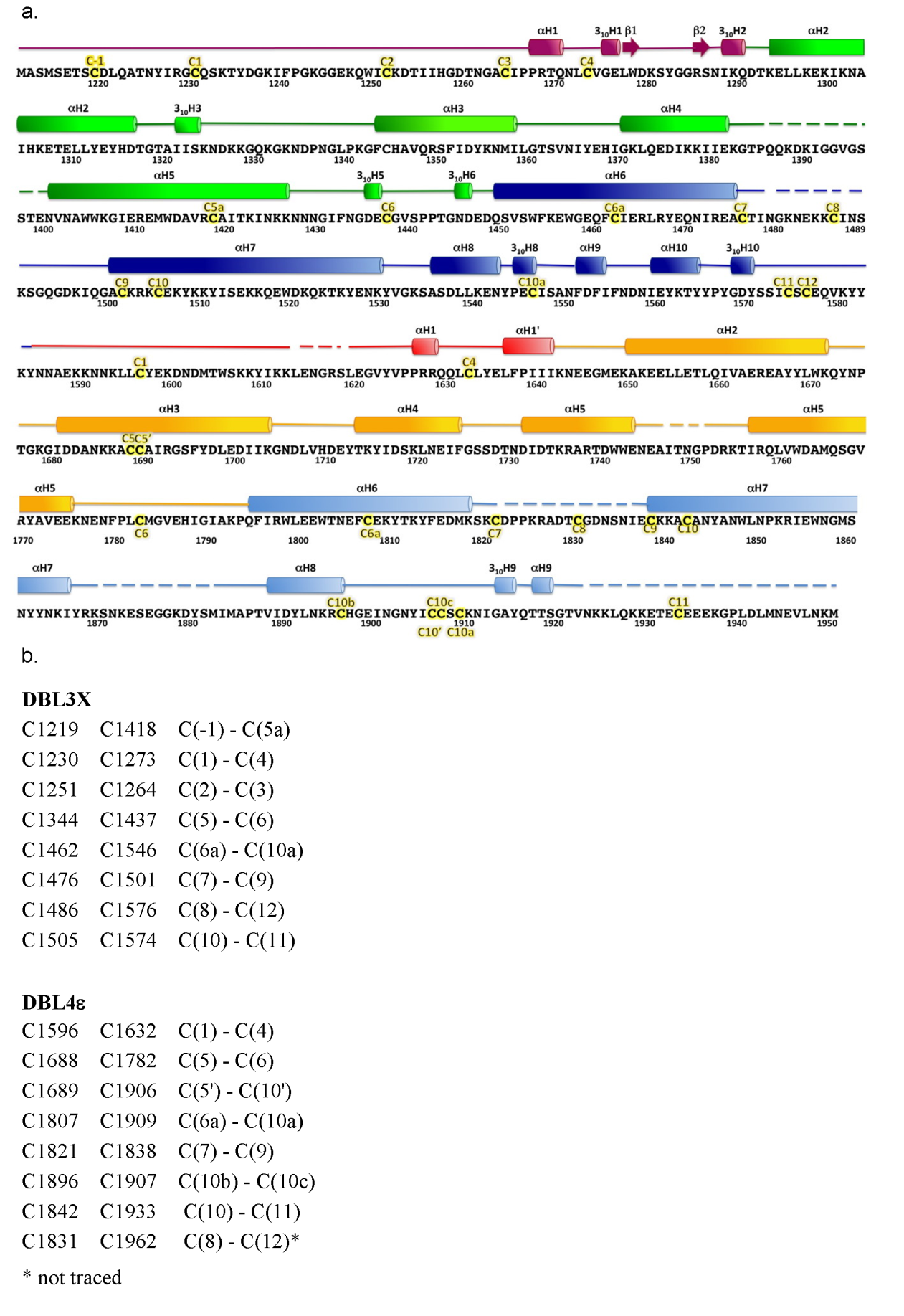
**

**Figure S1.** **Secondary structure of the FCR3-DBL3X-DBL4ε double domain.**

**a**. Helices are symbolised by cylinders, β-strands by arrows and numbered. Subdomains 1 are in mauve and red, Subdomains 2 in green and yellow, Subdomains 3 in dark and pale blue, for both DBL3X and DBL4ε. Regions of the polypeptide chain that were not traced in the electron density are indicated by dashed lines.

**b**. Residue numbers of cysteines forming a disulphide bridge are given in columns 1 and 2; the corresponding canonical cysteine numbering is given in columns 3 and 4.

**
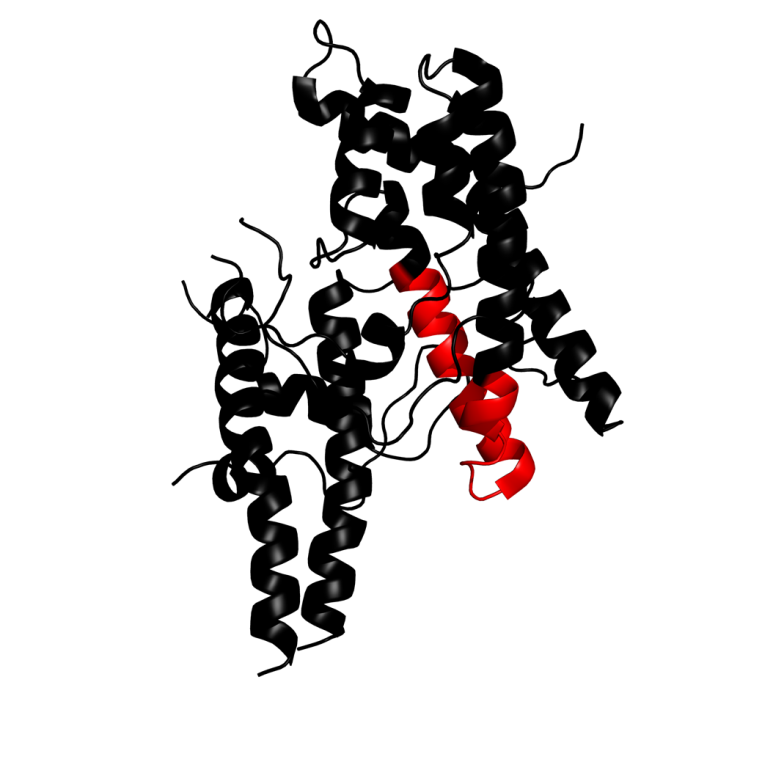
**

**Figure S2. DBL4ε region recognized by highly parasite blocking IgG.**

The highly antigenic DBL4ε region identified by Ditlev *et al.*, ^30^ is represented in red in the structural context of the single domain.

**
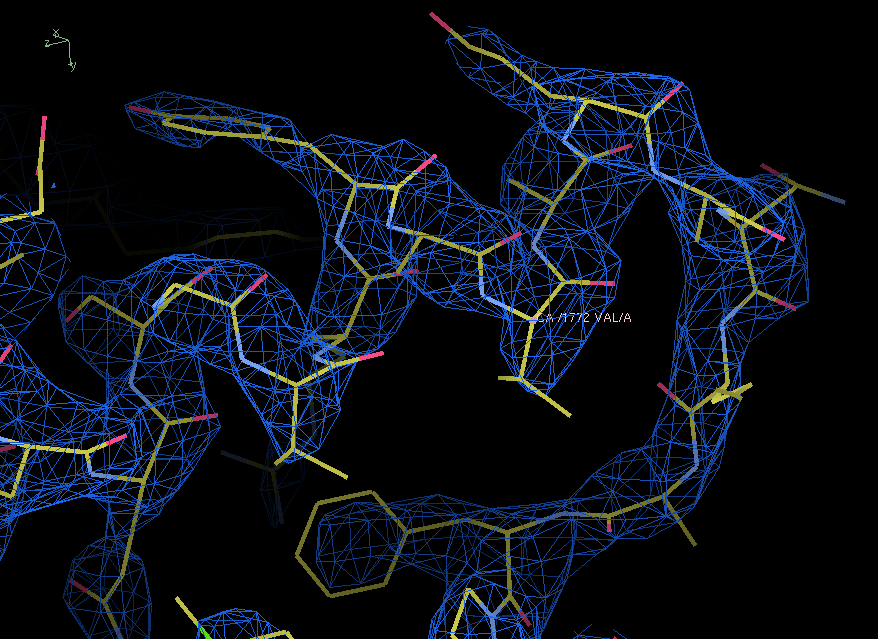
**

**
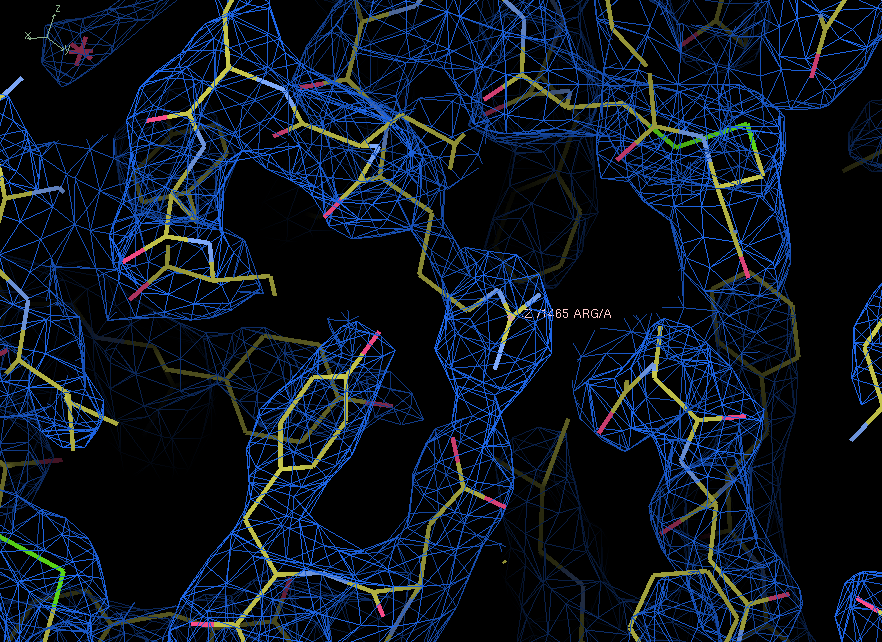
**

**Figure S3. Portions of Electron Density Map**

| **Domain** |  | **Contacts** | **Polymorphic** |
| --- | --- | --- | --- |
| **DBL3X** | **DBL4ε** | **Total (polar)** | **residues** |
|  |  |  |  |
| Arg1473 | Ile1641 | 5 (2) | - |
| ” | Lys1642 | 2 (1) | - |
| ” | Glu1644 | 6 (1) | DBL4ε: Lys/Glu/Asn |
| Ser1534 | Tyr1609 | 2 (1) | DBL3X: Ser/Phe |
| Ser1536 | Glu1711 | 1 | - |
| Asp1537 | Tyr1609 | 7 (1) | - |
| Ile1547 | Tyr1715 | 1 | - |
| Ser1548 | Phe1637 | 1 | - |
| ” | Tyr1715 | 2 | - |
| Asn1550 | Phe1637 | 2 | - |
| ” | Glu1711 | 4 (1) | - |
| ” | Tyr1712 | 6 | - |
| Asp1552 | Tyr1597 | 5 (2) | DBL4ε: His/Tyr |
| ” | Tyr1711 | 1 | - |
| ” | Tyr1712 | 2 (1) | - |
| Phe1553 | Tyr1712 | 2 | - |
| Asn1556 | Tyr1597 | 7 (1) | DBL4ε: His/Tyr |
| Glu1560 | Leu1595 | 1 | DBL3X: Glu/Asp |
| Tyr1561 | Leu1595 | 3 | DBL3X: Tyr/His |
| ” | Cys1596 | 1 | “ |
| ” | Tyr1597 | 1 | “ |
| ” | Tyr1634 | 4 | “ |
| Tyr1564 | Asn1592 | 3 | - |
| ” | Glu1635 | 2 | - |
| Tyr1565 | Glu1635 | 1 | - |
| ” | Arg1664 | 1 | - |
| Asp1569 | Pro1638 | 2 | - |
| ” | Ile1641 | 1 | - |
| Tyr1570 | Ile1641 | 2 | - |
| Ser1572 | Pro1638 | 3 (1) | - |
| ” | Ile1639 | 1 | - |
| ” | Lys1642 | 3 (1) | - |
| Ile1573 | Lys1642 | 2 | - |
| Val1579 | Ile1639 | 1 | - |
| ” | Lys1642 | 1 | - |
| ” | Glu1653 | 3 | - |
| ” | Thr1657 | 1 | - |
| Lys1580 | Glu1656 | 1 | - |
| Tyr1581 | Asn1591 | 6 | - |
| ” | Glu1656 | 4 (1) | - |
| ” | Gln1659 | 5 | DBL4ε: Gln/Lys |
| ” | Ile1660 | 1 | - |
| ” | Glu1663 | 5 (1) | - |
| ” | Ser1766 | 2 (1) | - |
| Tyr1582 | Asn1591 | 8 (2) | - |

**Table S1. Contacts at the DBL3X/DBL4ε interface.** The number of contacts (interatomic distances < 3.8 Å) and polar interactions is given in column 3 for pairs of residues of DBL3X (column 1) and DBL4ε (column 2). Polymorphic residues are indicated in column 4.
